# New Turns for High Efficiency Knock-In of Large DNA in Human Pluripotent Stem Cells

**DOI:** 10.1155/2018/9465028

**Published:** 2018-07-03

**Authors:** Xiangjun He, Yin-Xiong Li, Bo Feng

**Affiliations:** ^1^Key Laboratory for Regenerative Medicine, Ministry of Education, School of Biomedical Sciences, Faculty of Medicine, Chinese University of Hong Kong, Shatin, Hong Kong; ^2^SBS Core Laboratory, CUHK Shenzhen Research Institute, Shenzhen, China; ^3^Institute of Public Health, Guangdong Provincial Key Laboratory of Biocomputing, Key Laboratory of Regenerative Biology, Guangdong Provincial Key Laboratory of Stem Cell and Regenerative Medicine, South China Institute for Stem Cell Biology and Regenerative Medicine, Guangzhou Institutes of Biomedicine and Health, Chinese Academy of Sciences, Guangzhou, China; ^4^Guangzhou Institute of Biomedicine and Health, Chinese Academy of Sciences, Guangzhou 510530, China

## Abstract

The groundbreaking CRISPR technology is revolutionizing biomedical research with its superior simplicity, high efficiency, and robust accuracy. Recent technological advances by a coupling CRISPR system with various DNA repair mechanisms have further opened up new opportunities to overcome existing challenges in knocking-in foreign DNA in human pluripotent stem cells, including embryonic stem cells (ESC) and induced pluripotent stem cells (iPSC). In this review, we summarized the very recent development of CRISPR-based knock-in strategies and discussed the results obtained as well as potential applications in human ESC and iPSC.

## 1. Introduction

Successful isolation of embryonic stem cells (ESC) and reprogramming of somatic tissues into induced pluripotent stem cells (iPSC) significantly foster the stem cell research and development of regenerative medicine [1]. Given their robust capability of self-renewal and broad potentials to differentiate into all somatic lineages, human ESC and iPSC provide excellent tools for *in vitro* disease modeling and drug screening, as well as valuable cell sources for cell-based therapies [1]. To harness the full application potentials of human ESC/iPSC, targeted genome editing with high accuracy and efficiency has long been thought desirable. Hence, extensive and constant effort is made to develop relevant technology using various tools once they are available.

Back in the late 1980s, targeted genome editing through homologous recombination (HR) was first established in mouse ESC and then demonstrated in generating live mouse strains carrying predesigned genetic modification [2]. Despite its wide application, this approach requires laborious clonal expansions and sophisticated selections to identify the mouse ESC clones with correct modifications. Therefore, in human ESC and iPSC, which show intrinsically poor clonogenicity and inefficient homologous recombination, HR-based genome targeting as well as knock-in of large DNA have long been hindered.

Recent advent of engineered nucleases has opened new avenues to develop novel genome editing strategies. Zinc-finger nuclease (ZFN) [3], transcription activator-like effector nuclease (TALEN) [4], and type II prokaryotic clustered regularly interspaced short palindromic repeats (CRISPR)/CRISPR-associated 9 (CRISPR/Cas9) system [5, 6] have achieved great success in introducing site-specific DNA double-strand breaks (DSBs) with high accuracy and efficiency. In particular, the CRISPR/Cas9 system has rapidly gained popularity and becomes the most widely used tool, due to its superior simplicity and robust performance [7, 8]. In this system, a single guide RNA (sgRNA) forms a complex with Cas9 nuclease to recognize a variable 20-nucleotide target sequence adjacent to a 5′-NGG-3′ protospacer adjacent motif (PAM), thus introducing a DSB in the target DNA [6, 9].

With the application of engineered nucleases, DSBs are induced at selected target sites and then trigger various DNA repair processes, via the homology-directed repair (HDR) (termed HR previously), the nonhomologous end joining (NHEJ), or the recently identified microhomology-mediated end joining (MMEJ) pathways [10, 11]. Studies have exploited these diverse DNA repair mechanisms to develop various targeting strategies and introduce a broad range of genomic modifications [12, 13]. Importantly, the previous challenges to knock-in of large DNA in human ESC/iPSC has been addressed in recent studies, through establishing new targeting strategies coupled with the CRISPR/Cas9 system. In this review, we will focus on the very recent advances in developing novel targeting strategies for high efficiency knock-in of large DNA in human ESC/iPSC and discuss the remaining challenges and potential solutions.

## 2. New Development of HDR-Based Knock-In through Coupling to CRISPR/Cas9

**Figure 1:**
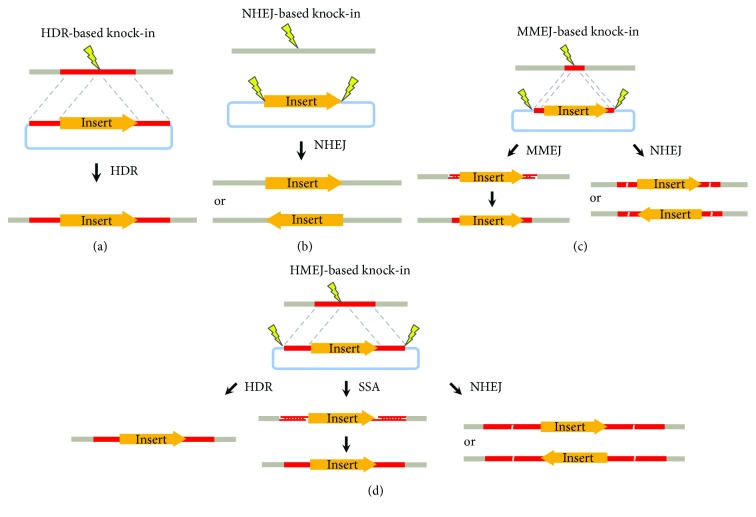
Schematic overview of HDR-, NHEJ-, MMEJ-, and HMEJ-based knock-in. (a) HDR-based knock-in requires long homology arms. (b) NHEJ-based knock-in, which requires the linearization of donor template rather than the homology arms. (c) MMEJ-based knock-in requires short homology arms (usually less than 50 bp). NHEJ-mediated knock-in might happen due to the presence of linearized donor template. (d) HMEJ-based knock-in requires the linearized donor template with long flanking arms. This method may trigger HDR-, SSA-, and NHEJ-based knock-in and yield combinatory outcome.

Influenced by the traditional gene targeting technology, engineered nucleases were first employed to enhance the HDR-based knock-in of foreign DNAs into the genome of human ESC/iPSC (Figure 1(a)). Hockemeyer et al. reported successful knock-in of reporter genes in human ESC/iPSC using short homology sequences (around 1 kb) at each side, through ZFNs in 2009 and TALEN in 2011 [14, 15], while Rong et al. and Merkle et al. reported the enhanced HDR-based knock-in using the CRISPR/Cas9 system [16, 17]. Since then, research has progressed rapidly to adopt technologies that have been established in the mouse system but was hindered previously in human ESC/iPSC due to the unavailability of genome editing tools. Chen et al. have developed an efficient two-step strategy to generate inducible knock-out of multiple genes in human ESC, through coupling CRISPR/Cas9 with the Flp/FRT and Cre/LoxP system [18]. Using Cas9 and sgRNAs driven by doxycycline-inducible promoter (iCRISPR), Zhu et al. demonstrated reporter knock-in at both active and silent loci in human ESC, without drug selection [19].

Despite the enhancement by CRISPR/Cas9 or other engineered nucleases, the HDR-based knock-in in human ESC/iPSC is still relatively inefficient [16]. Sophisticated selection scheme and cumbersome clonal expansion analysis, which are particularly tricky in human ESC/iPSC, are still required. Therefore, extensive investigations have focused to further improve the HDR-based knock-in efficiency. Along this trend, studies have sought further increase of the HDR-based knock-in efficiency in human ESC/iPSC, either by directly inhibiting the NHEJ pathway with small chemicals [20–22] or by enhancing HDR-based DNA repair through synchronizing cell cycles to the G2/M phase [23] or overexpressing RAD51 in the presence of valproic acid [24]. Moreover, studies also explore the potentials of surrogate reporters and showed that they could enrich the target human ESC/iPSC carrying HDR-based knock-in [25, 26].

On the other hand, the significantly enhanced HDR at a selected target site by the CRISPR/Cas9 system has enabled small DNA sequence replacement using short single-strand DNA as donors, which could be easily synthesized as single-strand oligodeoxynucleotides (ssODNs) [27]. This strategy is especially valuable to correct single point mutations, which are broadly associated with human diseases. Successful applications of ssODN have been demonstrated in various animal models through direct injection of CRISPR/Cas9 components into zygotes [28–30] or in human ESCs and iPSC for modeling human diseases [31–33]. Recently, with a step further, long single-strand DNA (lssDNA) has been employed for exogenous DNA knock-in through zygote injection [34]. This lssDNA-based knock-in demonstrated higher targeting efficiency than traditional HDR-based methods and is more suitable to generate large-scale Cre-LoxP animal resources [35]. Its potentials in knocking-in large DNA into human ESC/iPSC have not been explored.

Interestingly, other than correcting point mutations, studies have employed ssODNs to facilitate the genomic integration of large DNA fragments at a selected target site. Yoshimi et al. named it as “two-hit by gRNA and two oligos with a targeting plasmid” (2H2OP) [36]. In this system, nonhomologous large dsDNA fragments were integrated into specific genome locus, through a bridging process mediated by two ssODNs that share short homology sequences to both genome and donor DNAs. Using this strategy, Yoshimi et al. introduced the GFP cassette at mouse *Rosa26* locus where DNA break in genome was induced by CRISPR/Cas9 [36]. More significantly, large DNA replacement up to 58 kb and targeted insertion of BAC clone around 200 kb were successfully achieved using this 2H2OP method in rat zygotes [36]. It is interesting but remains to be confirmed whether the lssDNA-mediated or the ssODN-facilitated dsDNA-mediated HDR-based approaches are suitable for knock-in of large DNAs in human ESC/iPSC.

## 3. Orientating the NHEJ Pathway for the Knock-In of Large DNA

NHEJ and HDR are the two major pathways to repair DNA damage. While HDR repairs a broad range of DNA damages based on existing homology sequences, NHEJ is the primary mechanism to repair DSBs in mammalian cells, in a homology-independent manner. The NHEJ repair process is often accompanied with small deletions/insertions at the DSB repair junctions; thus, it is widely employed to introduce frame shift to generate gene knockout [37]. On the other hand, the NHEJ repair mechanism has long been found to mediate random integrations of exogenous DNA in host cell genome, which are widely used to generate transgenic animals or stable cells carrying ectopic gene expression [38]. However, the potentials of the NHEJ pathway in mediating knock-in of large DNA at a preselected target site have been largely overlooked, until the engineered nucleases were established recently.

Since 2010, two groups demonstrated successful knock-in of DNA fragments through generating sticky ends simultaneously in donor and genome DNAs via ZFN cleavage [39, 40]. Subsequently, a similar method was refined and named as ObLiGaRe [41]. The results obtained indicated that the NHEJ pathway could also facilitate exogenous DNA integrations, through ligating the blunt ends generated from ZFN- or TALEN-induced DNA cleavage in genome and donor DNA. This speculation has been further verified using the CRISPR/Cas9 system in lower vertebrates, such as zebrafish [42, 43] and *Xenopus* [44].

Remarkably, in 2016, He et al. conducted a systematic side-by-side comparison between the HDR- and NHEJ-based knock-in and demonstrated that the CRISPR/Cas9-coupled NHEJ approach was superior to the HDR-based knock-in strategy in all human cell lines examined, including human ESCs [45] (Figure 1(b)). Consistently, in a few months later, a study by Suzuki et al. also reported higher efficiency through NHEJ-based knock-in than HDR approaches, in human HEK293 cells as well as in live mice [46]. In addition, the direct quantitation using the promoterless reporter system in He et al.'s study revealed the efficiency of HDR-based knock-in in human ESC around 0.06–0.36%; whereas the knock-in via NHEJ-based strategies showed much higher efficiency, around 0.83–1.70% in human ESC [45]. These data demonstrated a significant improvement when compared with the previous studies where the efficiency of CRISPR-coupled HDR knock-in was estimated to be around 1 : 10^5^–10^6^ in human ESC/iPSC, through extensive clonal analysis [16].

On the other hand, consistent with previous studies [16, 47, 48], He et al. also showed that the knock-in efficiency in human ESC, via either pathway, is much lower than that observed in somatic cell lines [45], which suggest that human ESCs possess unique properties in repairing DNA damage. This has been puzzling, because DNA repair proteins were found to be highly expressed in human pluripotent stem cells [47, 48]. Consistently, recent studies in human preimplantation embryos showed that, after CRISPR/Cas9 induced DSBs at genome, DNA repair via either pathway is highly efficient, while NHEJ-induced indels were detected at higher frequency than the HDR-based repair events [49–51]. Interestingly, Ma et al.'s study further demonstrated that the mutant paternal allele was predominantly repaired using the homologous sequence in the wild-type maternal allele instead of the synthetic DNA template [50]. In contrast, they found that the efficiency of HDR in iPSC is much lower, and targeted DNA cleavage was primarily repaired based on the exogenous DNA template [50]. Apparently, further investigation is needed to clarify whether a unique DNA repair mechanism indeed exists in early human embryos and if DSB repair in human embryos and ESC/iPSC is regulated distinctly. This will provide new mechanistic insights into the unique DNA repair processes in early embryos and pluripotent stem cells, which, in turn, might allow to further alter the technology and improve genome editing in human ESC/iPSC.

## 4. A New Alternative to Knock-In via MMEJ Pathway

Other than HDR and NHEJ, the two major DNA repair pathways, recent studies have examined the potentials of the MMEJ repair pathway in mediating targeted knock-in of large DNAs. In 2014, Nakade et al. first showed that DNA integration could be efficiently achieved via MMEJ mechanism at a predefined locus using as short as 10 bp microhomology sequences, and they referred this method as PITCh [52] (Figure 1(c)). Besides cultured cell lines, successful applications of the MMEJ-based knock-in strategy have also been demonstrated in zebrafish, *Xenopus*, and mouse through zygote injection [52–54].

On the other hand, recent studies have reported comparisons among the different knock-in approaches mediated by HDR, NHEJ, and MMEJ repair pathways, in cultured mouse ESCs as well as primary astrocytes and neurons [55]. The MMEJ-based knock-in method provides unique advantage for the knock-in in nondividing cells, likely due to its high activity during G1/early S phase in the cell cycle. Similar comparisons have also been done under *in vivo* conditions through zygote injection or viral transduction in somatic tissues [46, 56]. However, the two studies employed distinct targeting strategies and performed the analysis in different cell contexts; the results obtained remain largely divergent from each other. Nevertheless, studies have not reported MMEJ-based targeting results in human ESC/iPSC up to date.

## 5. A Superior Combination of HDR and NHEJ-Based Knock-In in the Presence of CRISPR

Interestingly, other than exploiting HDR, NHEJ, or MMEJ repair mechanisms individually, a line of evidence suggests that a combinatory strategy may yield even better outcome in knocking-in large DNA. It was first reported in 2006 that targeted gene modification via ZFN-induced HDR was significantly enhanced by using an extrachromosomal linear donor in *Drosophila* [57]. Inspired by this study, Ochiai et al. demonstrated similar enhancement in the ZFN-mediated targeted insertion by in situ linearization of the targeting donor construct in sea urchin embryos [58]. Recently, studies further coupled this combinatory knock-in strategy to the CRISPR/Cas9 system, named HMEJ-based knock-in, and applied it in mouse ESC and human iPSC [55, 59]. In these studies, CRISPR/Cas9 was employed to induce DNA DSBs at two homology arms simultaneously in the donor and endogenous genome, thus providing a linear DNA fragment with long homology arms for subsequent HDR-based knock-in (Figure 1(d)). Interestingly, while the study in mouse ESCs by Yao et al. showed no significant improvement of the targeting efficiency, the investigation in human iPSC by Zhang et al. reported appealing increase of the targeted integration of large DNA fragments using this HMEJ strategy [55, 59]. He et al. have also examined the potential of linear DNA in HDR-based knock-in by linearizing the donor at either side of the homology-reporter-homology cassette. Interestingly, their study showed a drastic increase in targeted knock-in when the donor vector was linearized at 5′ end of the homology-reporter-homology cassette by CRISPR/Cas9 but not at its 3′ end [45]. It suggested that the drastically increased knock-in produced was likely a combinatory outcome of both NHEJ-based knock-in and HDR-based knock-in, in which, the single-strand annealing process might also be involved. It is likely that the high efficiency of HMEJ-based knock-in in studies by Yao et al. and Zhang et al. was achieved via the similar combinatory mechanisms [55, 59]. Nonetheless, further investigation is still needed to clarify the molecular events occurring during the HMEJ knock-in and to establish truly high-efficiency knock-in of large DNA in human ESC and iPSC.

## 6. Perspectives

The field of genome editing is rapidly evolving due to fast development of new technologies. Recent advances in various CRISPR-based knock-in strategies have opened up new opportunities to overcome current challenges, and further research on this direction is highly promising to achieve high-efficiency genome editing in human ESC/iPSC. This will promote development of more simplified and cost-effective technical procedures to correct disease causative mutations in patient-derived iPSC or to introduce these mutations in human ESC, which will further improve the understanding of relationships between genetic mutations and perturbations in various cellular functions [32, 60]. Meanwhile, the high-efficiency genome editing in human ESC/iPSC will also provide cell-based platforms, which could establish new insights into the molecular basis of differentiation or facilitate drug screening [61]. The recent success in deriving organoids from human ESC/iPSC has provided more advanced models by generating tissues “in a dish” [62, 63]. Combining this organoid technology and the high-efficiency genome editing in human ESC/iPSC will provide a fascinating tool, which will be highly powerful to further promote study of developmental processes, tissue-based function, or pathological progress related to specific genetic defects [64, 65]. Altogether, it is foreseeable that applications of the newly developed gene targeting strategies will significantly boost the research on human ESC/iPSC and promote the progress of utilizing human pluripotent stem cells in regenerative medicine.
